# Validating a Consumer Smartwatch for Nocturnal Respiratory Rate Measurements in Sleep Monitoring

**DOI:** 10.3390/s23187976

**Published:** 2023-09-19

**Authors:** Hyunjun Jung, Dongyeop Kim, Jongmin Choi, Eun Yeon Joo

**Affiliations:** 1 Samsung Electronics, Suwon 16677, Republic of Korea; 2 Department of Neurology, Ewha Womans University Seoul Hospital, Ewha Womans University College of Medicine, Seoul 07804, Republic of Korea; 3 Departments of Neurology, Samsung Medical Center, Sungkyunkwan University School of Medicine, Seoul 06351, Republic of Korea

**Keywords:** wearable device, respiratory rate, photoplethysmography, accelerometer, obstructive sleep apnea, sleep monitoring

## Abstract

Wrist-based respiratory rate (RR) measurement during sleep faces accuracy limitations. This study aimed to assess the accuracy of the RR estimation function during sleep based on the severity of obstructive sleep apnea (OSA) using the Samsung Galaxy Watch (GW) series. These watches are equipped with accelerometers and photoplethysmography sensors for RR estimation. A total of 195 participants visiting our sleep clinic underwent overnight polysomnography while wearing the GW, and the RR estimated by the GW was compared with the reference RR obtained from the nasal thermocouple. For all participants, the root mean squared error (RMSE) of the average overnight RR and continuous RR measurements were 1.13 bpm and 1.62 bpm, respectively, showing a small bias of 0.39 bpm and 0.37 bpm, respectively. The Bland–Altman plots indicated good agreement in the RR measurements for the normal, mild, and moderate OSA groups. In participants with normal-to-moderate OSA, both average overnight RR and continuous RR measurements achieved accuracy rates exceeding 90%. However, for patients with severe OSA, these accuracy rates decreased to 79.45% and 75.8%, respectively. The study demonstrates the GW’s ability to accurately estimate RR during sleep, even though accuracy may be compromised in patients with severe OSA.

## 1. Introduction 

Measurement of the respiratory rate (RR), a vital sign, is an important physiological indicator. In particular, RR during sleep can be used to estimate sleep stage by observing the variations in the RR throughout a single sleep session [1,2]. Moreover, RR assessment has proven valuable in detecting clinical conditions, including COVID-19 infection [3] and exacerbation of chronic obstructive pulmonary disease [4], and serving as an indicator of acute coronary syndrome [5]. 

Numerous techniques have been developed to measure RR during sleep [6]. These techniques include use of breathing sounds generated at the nasal and oral levels through airflow [7], breathing motion detected using radar-based techniques [8], modulation in photoplethysmography (PPG) caused by respiratory sinus arrhythmia [3], motion captured by wrist-mounted accelerometers (ACCs) and gyroscopes [9,10], or optical measurements obtained from observation of the chest [11]. Among these techniques, PPG-based technology can be widely applied because these sensors are installed in many wearable devices and already record various biometric functions such as heart rate [12], peripheral oxygen saturation [13], and sleep stage [14]. Despite the ACC-based method being intuitive and simple to integrate, most researchers are focusing on patch-type devices attached to the chest [15] for obtaining large respiratory motion signals. Only a few studies have shown the feasibility of wrist-based measurements. 

However, measuring RR during sleep at the wrist is challenging. The accuracy of PPG-based measurements depends on the chosen location, among which the wrist is generally considered to have relatively low accuracy [16]. PPG-based techniques are limited in their ability to accurately measure RR during deep breathing, particularly when the rates are very low [17]. In addition, ACC-based measurements are influenced by arm position; there may be no signal when the arm is away from the body. Furthermore, there is a lack of information regarding the accuracy of RR measurements based on sleep posture and uncontrollable use, which can distort PPG signals. Specifically, the noise associated with obstructive sleep apnea (OSA) can significantly impact RR estimation by modulating PPG and ACC signals. Consequently, some previous studies classified apnea and hypopnea epochs as artifacts, excluding them from the analysis [3,16]. It is important to characterize the effect of nonbreathing periods on RR estimation when measuring on using wrist. In this study, we aimed to evaluate the accuracy of RR estimation during sleep using a wrist-worn device that combines both PPG-based techniques and micro-motion extracted from an ACC to enhance the performance of RR estimation. 

## 2. Methods 

### 2.1. Device under Test

Our target devices were the Samsung Galaxy Watch 4 and 5 series (GW, Samsung Electronics, Seoul, South Korea), both of which incorporate sensors and algorithms with the same performance for RR measurements. The GW is a smartwatch that allows users to track their fitness and health using various sensors, including an accelerometer (ACC), a gyroscope, photoplethysmography (PPG), and GPS. The watch offers the tracking of several biometric signals, including heart rate, electrocardiogram, blood oxygen level, and sleep. While the GW does not offer direct RR values, raw ACC and PPG data can be accessed through the Privileged Health Software Development Kit v1.2.0 (https://developer.samsung.com/health/privileged), enabling RR estimation with a dedicated algorithm. The RR estimation algorithm is not available for commercial use at the moment. This study evaluated the accuracy of the process of acquiring physiological signal data during sleep using a wrist-worn device and estimating RR through appropriate signal-processing methods can be performed. Both ACC and PPG signals were sampled at a frequency of 25 Hz and stored within the device through a customized application. Among the three emitted lights constituting the PPG sensor, a green signal with a wavelength of 525 nm was chosen for RR data collection due to its high sensitivity to changes in blood flow, high signal-to-noise ratio, and high optical absorption capabilities by hemoglobin. 

### 2.2. Participants and Study Procedure

The participants were recruited from among the adult patients who visited the sleep clinic at Samsung Medical Center, Seoul, South Korea, who had complaints of sleep disturbances and were undergoing polysomnography (PSG). After receiving detailed explanations about the study procedures, all participants provided written informed consent. Excluding the 5 with PSG sensor errors, a total of 195 participants were enrolled. Participants underwent overnight polysomnography (Embla N7000, Medcare Flaga, Reykjavik, Iceland) while wearing the GW on the nondominant arm. The RR data from the PSG and GW were collected from when lights were turned off until the participants awoke and turned on the lights. Episodes of apnea and hypopnea were defined according to the American Academy of Sleep Medicine guidelines, and participants were classified into the following categories based on apnea-hypopnea index (AHI): no OSA (AHI < 5/h), mild OSA (5/h ≤ AHI < 15/h), moderate OSA (15/h ≤ AHI < 30/h), and severe OSA (AHI ≥ 30/h). The study protocol received approval from the Institutional Review Board of Samsung Medical Center (IRB nos. 2021-04-166 and 2022-08-029).

### 2.3. Signal Processing Method

To extract the periodicity of the PPG and ACC data from the watch, we used three PPG-derived features (respiratory-induced frequency variation (RIFV), respiratory-induced amplitude variation (RIAV), and respiratory-induced intensity variation (RIIV)) [18] and three ACC-based features (ACC X, Y, and Z). We removed the baseline noise from the PPG signal using a 0.1–1.5 Hz bandpass filter and then detected each beat using a modified slope-based algorithm [17]. After detecting each beat from the PPG signal, we extracted the interval and amplitude features from the filtered PPG, while feature intensity was obtained from the raw PPG signal. These features were then stored in a buffer for spectrum estimation during a specific time period. Then, the spectrum was calculated using a Lomb–Scargle periodogram because of the uneven sampling of the data. Similarly, the ACC signal was bandpass-filtered using a 0.1–1 Hz filter, and the spectrum was estimated using a one-minute window. Through these steps, six estimated spectra from different features were stored in a buffer for a certain period. These spectra were then merged into a single spectrum, from which the main frequency representing the RR was estimated. When valid peaks were present in the merged spectrum, RR values could be calculated. Here, ‘valid peaks’ referred to peaks located within a certain range of the previous RR value. If valid peaks were found, we calculated the RR using peak location and updated it through a weighted average of the previous RR. However, if no valid peaks were detected, due to artifacts derived from participants’ motion or poor device–skin interface, there was no available RR value. In such cases, the previously stored RR value in the algorithm was used it to determine whether the subsequently appearing peak was valid. To ensure robustness, the RR-value-tracking method was used to avoid miscalculations during low-quality-signal periods. The estimated RR from the GW was updated every minute, and this was defined as the watch RR in this study.

The periodicity from the nasal thermocouple was extracted to collect RR data from the PSG, defined as the reference RR. A 0.1–0.6 Hz bandpass filter was used to eliminate noise and baseline drift, and the zero-crossing rate was calculated using a five-minute window with a one-minute shift. To detect periods of missing data, we calculated the variation in a 10-s window and excluded the window periods in which the variation was zero.

### 2.4. Validation Analysis

The performance of the watch was assessed through comparison with the reference RR using root mean square error (RMSE) and bias. RMSE measures the average magnitude of the differences between the estimated RR and the reference RR. A lower RMSE indicates higher accuracy, as this signifies smaller discrepancies between the two sets of data. Bias quantifies the systemic error of the estimation of RR from measurements by the watch. The accuracy was determined by calculating the percentage of RR estimates from the watch within the range of two breaths per minute (bpm) based on the reference RR. A Bland–Altman plot was employed to provide visual representation of the agreement between the watch RR and reference RR. The RR measurement method was validated through two approaches: calculation of the average RR over the entire night and continuous RR measurements collected in one-minute intervals. Then, we assessed how the performance of RR estimation varied across OSA severity groups. The performance evaluation was conducted using MATLAB version R2020a (The MathWorks Inc., Natick, MA, USA).

## 3. Results 

### 3.1. Participants

The demographics and polysomnographic findings of the participants are summarized in Table 1. 

### 3.2. Breathing Signals Captured by a Wrist-Worn Device

**Figure 1:**
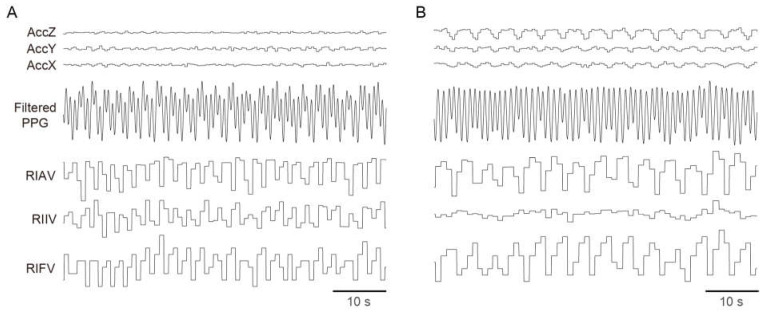
Representative traces of accelerometer- and photoplethysmography-derived breathing signals from a wrist-worn device, when the device was far away (**A**) or close (**B**) to the trunk.

Breathing signals were measured during sleep using a wrist-worn ACC, and this measurement was dependent on the sleeping posture (Figure 1). The ability to extract periodicity from the ACC signals varied depending on the position of the watch relative to the trunk, particularly in terms of distance. For example, large breathing signals could be captured when the watch-worn hand was positioned above the abdomen (Figure 1B), but there were no breathing signals detected when the hand was located far from the trunk (Figure 1A). The breathing signals from the ACC were captured independently for the three axes (X, Y, and Z axes). Similar to the ACC signals, the degree of modulation in the interval (RIFV), amplitude (RIAV), and baseline (RIIV) differed. However, during the same period, breathing features from the PPG signals showed synchronized modulation with the accelerometer in both cases. Consequently, we inferred that the PPG and ACC signals could provide robust RR estimation when used complementarily. The spectra from both PPG and ACC signals were weighted equally. The algorithm then selected those spectra with valid spectral peaks, allowing us to exclude features without respiratory signals and increasing the likelihood of selecting spectra with a stronger respiratory signal. The ACC signals offered higher temporal resolution than the PPG signals because the temporal resolution of PPG-derived breathing features is dependent on the heart rate. In addition, for such cases as those presented in Figure 1B, the ACC signals exhibited a higher signal-to-noise ratio, leading to a higher priority for ACC-based features in the RR estimation algorithm. Conversely, although the temporal resolution of the PPG signals was lower than that of the ACC signals, the PPG-based signals offered good quality -breathing features under proper use conditions, even when the ACC signals were poor. In such cases, PPG-based features were adopted with a higher priority in the RR estimation algorithm. Furthermore, low-frequency and high-amplitude physiological artifacts, which appear to be Mayer’s wave [19], were observed. Hence, our algorithm employs complementary use of six features (RIFV, RIAV, RIIV, ACCX, ACCY, and ACCZ) to address these limitations.

### 3.3. Respiratory Rate Measurements during Sleep 

**Figure 2:**
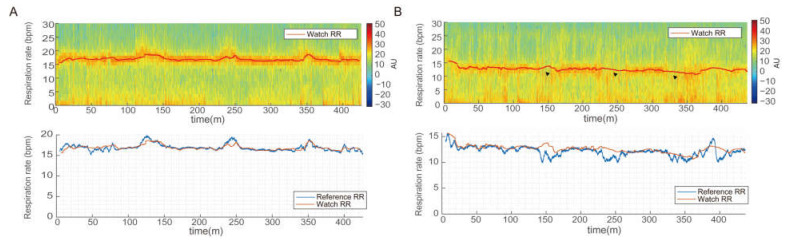
Examples of comparison of estimated respiration rate from the wearable device with the reference derived from nasal thermocouple of polysomnography in patients with mild (**A**) and moderate (**B**) obstructive sleep apnea. Reference respiration rate (RR) is represented in two ways: the log-scaled time-frequency analysis spectrum (**upper** panel) and a line depicting zero-crossing rate using a five-minute window with a one-minute shift (**lower** panel). In mild OSA (**A**), a continuous high-power band appears in the spectrogram with high-accuracy RR estimation by the watch. However, in moderate OSA (**B**), there is a weak power band and large variability in the spectrogram (arrowheads) with low-accuracy RR estimation.

The distribution of RR during sleep was about 10–22 bpm, which is similar to the results reported in a previous study [3]. Figure 2 presents a comparison between the watch RR and the reference RR for two scenarios. In a patient with mild OSA with an AHI of 6.9, a continuous high-power band appeared over time in the spectrogram, and this band was followed closely by the reference RR and watch RR (Figure 2A). However, in a patient with moderate OSA with an AHI of 26, the power was weak and the variability was large in the spectrogram (Figure 2B). The reference RR was calculated during the apnea period to account for the utilization of long-window-length data (five minutes) in the spectrum-based peak-frequency estimation, which could include normal breathing periods. During the apnea period, PPG signals may exhibit respiration-related features [20,21], and ACC signals may capture chest wall motion [22]. In addition, the watch RR output was obtained due to the use of a one-minute window and the tracking algorithm employed in the spectrum-based peak frequency estimation. This one-minute window had a high likelihood of encompassing normal breathing periods.

### 3.4. Comparison of Average Overnight Respiratory Rate

**Figure 3:**
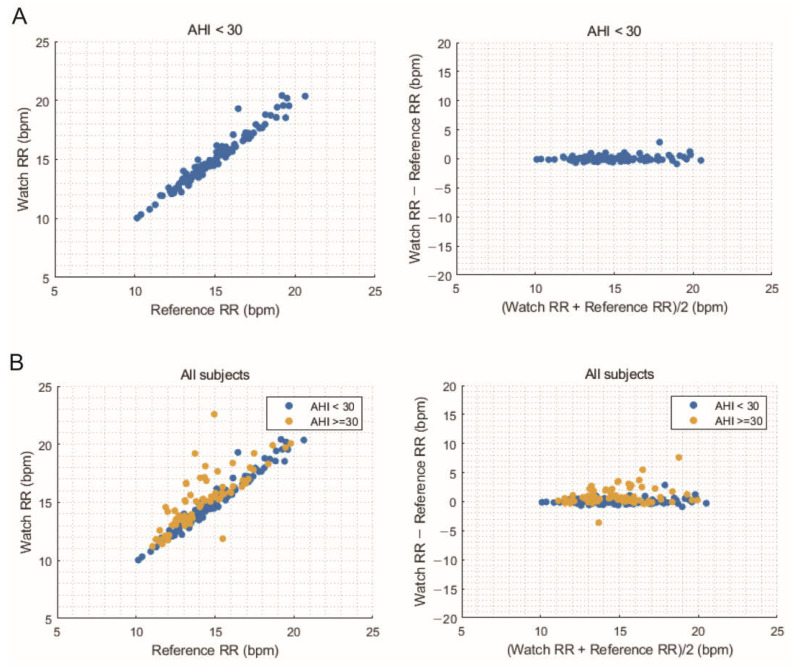
Correlation and Bland–Altman plots comparing the average overnight respiration rate between the watch and reference for the apnea-hypopnea index < 30 group (**A**) and all subjects (**B**).

To evaluate the performance of the GW, the average RR measured using the PSG and GW throughout the night was compared (Table 2). The RMSE calculated across all subjects was 1.13 bpm with a bias of 0.39 bpm. However, accuracy improved to an RMSE of 0.46 bpm, with a bias of 0.08 bpm for the subgroup of participants with an AHI < 30. The accuracy of the estimated average overnight RR using the GW was 99.18% for participants with an AHI < 30, whereas it was 91.79% for all subjects. The GW tended to overestimate the RR for participants with higher numbers of apnea and hypopnea events, resulting in a positive bias. These findings are further demonstrated by the correlation and Bland–Altman plots (Figure 3), which show strong agreement for participants with an AHI < 30, while plots for all subjects demonstrate a slightly reduced level of agreement. Moreover, the Bland–Altman plot indicates a systematic bias that increases as RR values increase.

### 3.5. Comparison of Continuous Respiratory Rates

**Figure 4:**
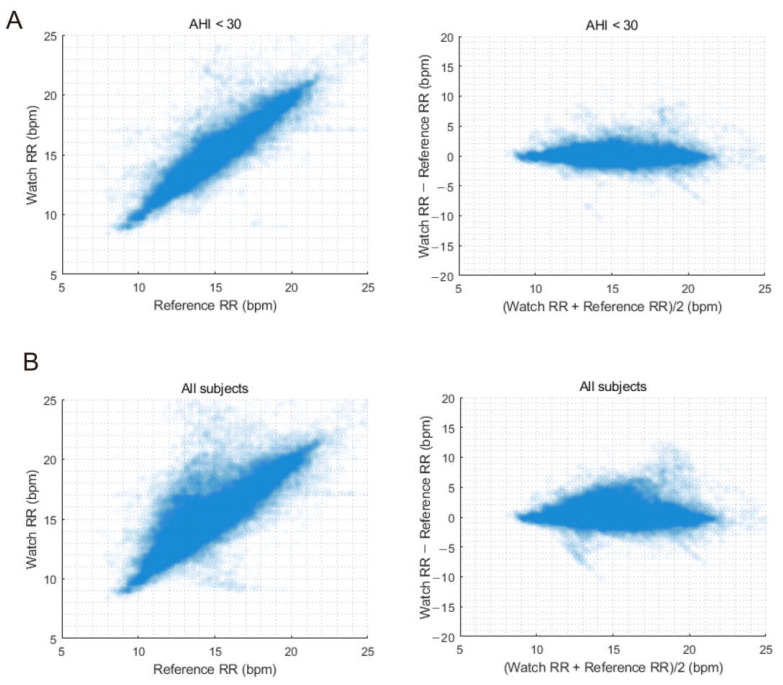
Correlation and Bland–Altman plots comparing continuous respiration rate measurements between the watch and reference for apnea-hypopnea index < 30 group (**A**) and all subjects (**B**).

We evaluated the performance of watch-recorded RR for continuous measurements after excluding data points with invalid one-minute RR values, which were unmeasurable during the initial stabilization period or due to artifacts. For participants with an AHI < 30, the RMSE of the watch RR was 1.22 bpm, with a bias of 0.08 bpm and an accuracy of 92.63%. The RMSE was 1.62 bpm, with a bias of 0.37 bpm and an accuracy of 86.66% for all subjects (Table 3). These results show a slightly lower performance compared with that based on the average overnight RR. However, for participants with an AHI < 30, the accuracy remained high, over 90%. Moreover, the overall performance was excellent, with an RMSE ± 2 bpm for all subjects. Similar correlation and agreement results for average overnight RR are shown in Figure 4, where including patients with more severe apnea leads to larger dispersion and lower accuracy in estimating RR.

### 3.6. Estimation of Respiratory Rate According to Obstructive Sleep Apnea Severity 

**Figure 5:**
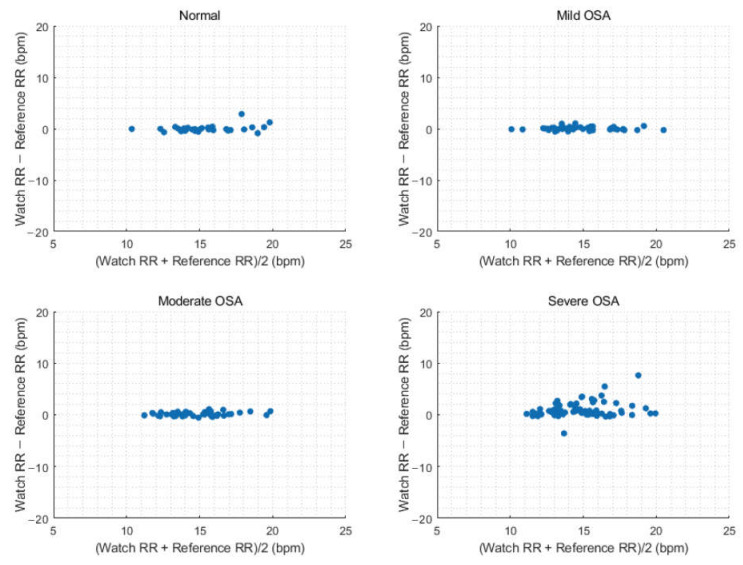
Bland–Altman plots comparing the average overnight respiration rate depending on obstructive sleep apnea severity.

**Figure 6:**
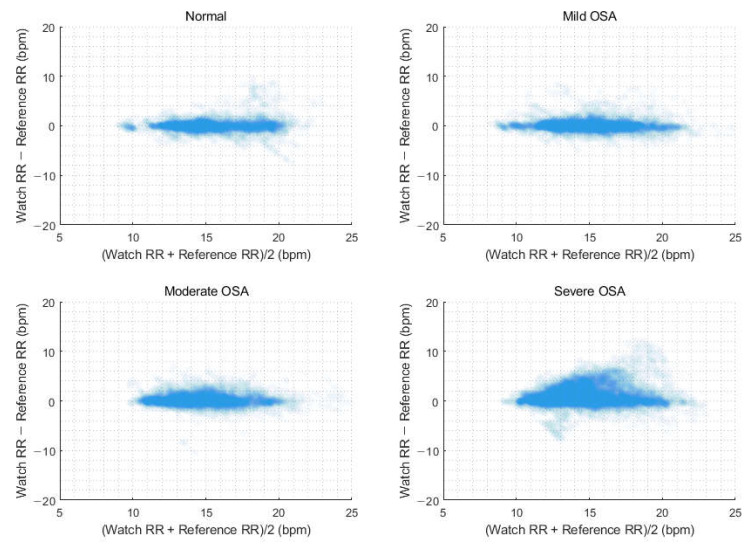
Bland–Altman plots comparing the continuous respiration rate measurements depending on obstructive sleep apnea severity.

Next, we evaluated performance of watch-recorded RR depending on AHI severity (Table 4). The average overnight watch RR showed optimal performance for normal, mild, and moderate OSA groups, with RMSE values of 0.64, 0.36, and 0.39 bpm and accuracy values of 96.77%, 100%, and 100%, respectively. In the severe OSA group, the RMSE increased to 1.74 bpm, and the accuracy decreased to 79.45%. Overall, the accuracy of continuous RR measurements was lower than that of the average overnight RR. Specifically, for the normal, mild, and moderate OSA groups, the RMSE values were 1.33, 1.26, and 1.15 bpm, with corresponding accuracy values of 92.39%, 92.61%, and 92.50%, respectively. In the severe OSA group, there was an increasing tendencies of overestimation and dispersion of the RR by the GW as RR increased; the RMSE was 1.74 bpm, and the accuracy was 79.45%. Figure 5 and Figure 6 display Bland–Altman plots for average overnight RR and continuous RR measurements, respectively, by OSA severity.

## 4. Discussion

In this study, we found that the GW can accurately measure the nocturnal RR using complementary PPG and ACC information. By prioritizing features with a stronger respiration signal quality, the RR estimation algorithm employs a complementary approach for feature selection. The average overnight RR exhibited an RMSE of 1.13 bpm and a bias of 0.39 bpm for all subjects who visited the sleep clinic with sleep problems. In addition, regarding the performance of continuous RR measurements, the RMSE was 1.62 bpm with a bias of 0.37 bpm. In the group with an AHI of less than 30, the RMSE of the average overnight RR ranged from 0.36 to 0.64 bpm, with an accuracy greater than 96%, while the RMSE of the continuous RR measurements ranged from 1.15 to 1.33 bpm, with an accuracy exceeding 92%. We demonstrated that frequent apnea and hypopnea events, especially those occurring at a rate of more than 30 times per hour, are important factors that affect the accuracy of RR measurements using wearable devices during sleep. For all subjects, the GW demonstrated high accuracy in RR estimation, indicating that additional data processing, such as OSA period detection, may not be necessary. Nevertheless, it is noteworthy that accuracy may be compromised when specifically targeting severe OSA patients. Further research and algorithm improvement are necessary to provide precise RR estimations in severe OSA cases, or it might be possible to predict severe OSA cases using the PPG signal and inform the user when accuracy is compromised in such instances. 

Our results demonstrated good performance that was comparable to that of previous methods. Previous studies using wrist-worn devices for estimating RR during sleep are limited. In a study utilizing the SleepMonitor system, three-axis ACC data were collected continuously through the night; a multiaxis fusion approach was applied to detect subtle periodic movements transmitted through the wrist during respiration, allowing measurement of RR and body position [9]. The majority of the data points in the Bland–Altman plot were within the range of ±1.96 standard deviations, and the mean absolute error from the reference values was 0.0274 bpm, with a standard deviation of 1.0235 bpm. In another study employing Fitbit for RR estimation, the researchers calculated the average over the night and reported favorable performance; the RMSE was 0.648 bpm, and the mean absolute error was 0.46 bpm [3]. However, patients diagnosed with severe OSA were excluded from the study.

Various types of devices, other than wearable devices worn on the wrist, have been utilized for estimating RR during sleep. These devices can be broadly categorized into two methods: those attached to the body and use ACC data [10,15,23] and those that employ contactless methods such as radar, sound, or ballistocardiogram signals [7,8,24,25]. In a previous study by Javier et al. [10], they showed the feasibility of motion-based RR estimation using ACC and gyroscope measurements with a watch-type wearable device in participants with unknown OSA status, achieving an RMSE ranging from 1.25 to 2.4 bpm, which is comparable to our result. Other motion-based RR estimation methods have focused on measuring breathing signals from the chest and abdomen, potentially yielding better signal quality than that from the wrist [15,23]. Wearable devices and contactless devices yielded similar accuracy, but the wearable device had fewer measurement errors and showed the ability to measure extreme values relatively accurately [26]. Among these, in a previous study using motion analysis with a near-infrared camera for estimating RR and heart rate, the accuracy of RR estimation decreased when apnea or hypopnea occurred compared with the normal 30-s time window during sleep [25]. Moreover, similar to our results, this study confirmed that the RMSE of the RR remained relatively consistent from normal to moderate OSA but exhibited a significant decline in accuracy in severe OSA cases.

Challenges in measuring RR during sleep, compared with wakefulness, include the intricacy caused by unconscious movements, resulting in movement artifacts. Also, changes in the position of wearable devices create difficulties in measuring PPG signals or detecting regular movements associated with respiration. In addition, the occurrence of hypopnea or apnea can contribute to an increase in errors in RR estimation. During apnea episodes, the PPG and ACC modulation signals lose their signal power, resulting in lower-accuracy periodicity estimation. Another possible explanation for the low accuracy observed in severe OSA patients is that the PPG-based method relies on respiration sinus arrhythmia, and the modulation of PPG signals by respiration may be minimal in severe OSA patients [23]. These factors collectively pose challenges for accurate RR estimation during sleep, particularly in cases of severe OSA.

The reason that the watch and reference RR were calculated during an apnea period is that our RR estimation approach relies on spectral peak analysis. The RR can be estimated by dividing number of breathing events by duration, and this measurement results in a lower RR when the apnea period is included in the calculation. However, as our RR estimation is based on frequency-domain analysis, the resulting output represents the maximum RR observed within the designated window period, regardless of the presence of apnea periods. Despite the presence of apnea and hypopnea periods, the inclusion of modulated PPG and ACC signals in the analysis is possible; the dominant modulation frequency derived from these signals may have a significant impact on RR estimation [20,21]. 

The GW overestimated the RR compared with the reference value, and there was a tendency for the watch RR to exhibit an increase in error as AHI increased. For participants whose AHI was less than 30/h, the mean error was negligible, measuring 0.01–0.15 bpm. However, in individuals with severe OSA, a noticeable increase in error was observed. The RMSE was 1.74 bpm, with an error of 0.93 bpm. Both ACC and PPG data may contribute to increased errors in patients with severe OSA. We assumed that the reason is the different temporal dynamics of the RR estimations. The reference RR employs a long window length of five minutes, while the watch RR uses a tracking algorithm that assigns a high weight value to previous outputs. Therefore, the watch RR moves much slower than the reference RR, as described in Figure 2B, resulting in an error between the watch RR and reference RR. In addition, partial or complete airway obstruction can affect the reference RR obtained through flowmetry, causing the RR to decrease. However, the watch RR, which captures abdominal and thoracic movements using ACC data even in the presence of airflow obstruction, estimates the respiratory rate and may result in overestimation. Furthermore, desaturation or arousal commonly accompanies the occurrence of apnea or hypopnea, followed by observations of movement and sympathetic overactivity. When the wearer moves, ACC and PPG data quality can be affected, regardless of actual respiration. As we demonstrated in our previous study, just as the accuracy of measuring oxygen saturation through PPG decreases with increasing severity of sleep apnea, the accuracy of RR estimation through PPG can also decrease due to poor periodicity in the features derived from the ACC and PPG [13].

There were several limitations in estimating the RR with a wrist-worn device. First, performance depends considerably on the use conditions of the watch because acquisition of a reliable PPG signal from the wrist requires proper device placement and a well-established device-to-skin interface, including appropriate pressure. Second, when the hand wearing the watch is positioned far from the body, respiration-derived movements may not be effectively transmitted to the wrist, leading to a decrease in the accuracy of RR estimation. Unfortunately, during sleep, individuals cannot consciously rectify this issue. Third, RR estimation was conducted using a relatively long one-minute time window, which moved slowly with a high weighing factor. Previous research using RR for sleep staging suggested the need for shorter time epochs (30 s) to capture the high RR variability during rapid-eye-movement sleep. However, due to concerns about accuracy and tradeoff considerations, we could not reduce the time window in this study [1].

Despite these limitations, measuring RR during sleep with a wearable device offers several advantages. As personalized life-logging tools, these devices can provide health assistance and detect abnormalities using past information. In particular, with the increasing interest in sleep health, various companies are incorporating features that assess sleep quality using wearable devices of different form factors. Among these, watch-type wearable devices are already widely adopted due to their high convenience and accessibility. These devices can provide valuable insights, including regarding sleep stages, oxygen saturation during sleep, and breathing rates, all of which are obtained from built-in sensors. By combining RR data with various existing biological signals, there is great potential to enhance the quality of the health information obtained.

## 5. Conclusions

In this study, the accuracy of RR measurements during sleep was evaluated using a wrist-worn wearable device. The results showed that the average overnight RR and continuous RR measurements demonstrated high accuracy, though accuracy may be compromised in patients with severe OSA. The achieved level of accuracy for continuous measurement of RR during sleep holds promise for enhancing sleep health assessments and contributing to better sleep management. In addition, monitoring the RR may be beneficial in the early detection of changes in breathing patterns and in detecting significant deviations from the normal respiratory pattern. Further research into the characteristics of the respiratory patterns may offer possibilities for apnea prediction.

## Figures and Tables

**Table 1:** Baseline characteristics of the participants.

Variable	All (*n* = 195)	AHI < 30 (*n* = 122)	AHI ≥ 30 (*n* = 73)	*p*-Value
Demographics				
Age, years	48.9 ± 13.3	47.6 ± 13.9	51.1 ± 12.1	0.067
Male, n (%)	143 (73.3)	78 (63.9)	65 (89.0)	<0.001
BMI, kg/m^2^	25.9 ± 4.0	24.7 ± 3.6	28.1 ± 3.8	<0.001
Polysomnographic parameters				
TST, min	339.7 ± 61.9	350.2 ± 59.0	322.1 ± 63.0	0.002
Sleep latency, min	11.5 ± 13.0	11.5 ± 12.5	11.4 ± 13.8	0.966
WASO, %	16.5 ± 11.8	15.8 ± 11.9	17.8 ± 11.6	0.265
Sleep efficiency, %	81.3 ± 12.2	82.0 ± 12.1	80.0 ± 12.4	0.283
N1/TST, %	20.5 ± 12.1	15.4 ± 6.9	28.9 ± 14.1	<0.001
N2/TST, %	53.1 ± 11.2	56.1 ± 9.9	48.2 ± 11.6	<0.001
N3/TST, %	6.9 ± 8.7	8.3 ± 9.6	4.4 ± 6.5	<0.001
REM/TST, %	19.6 ± 6.7	20.2 ± 6.0	18.5 ± 7.7	0.106
AHI, /h	28.5 ± 24.9	12.3 ± 8.4	55.6 ± 19.2	<0.001
Total AI, /h	29.3 ± 17.3	20.7 ± 7.7	43.5 ± 19.4	<0.001
Respiratory AI, /h	19.2 ± 20.0	7.7 ± 5.9	38.4 ± 20.5	<0.001
Lowest saturation, %	84.0 ± 8.4	87.5 ± 5.3	78.0 ± 9.2	<0.001

AHI, apnea-hypopnea index; BMI, body mass index; TST, total sleep time; WASO, wakefulness after sleep onset; N, nonrapid eye movement sleep; REM, rapid eye movement sleep; AI, arousal index.

**Table 2:** Average overnight respiratory rate estimation performance.

Parameter	AHI < 30 (*n* = 122)	All Subjects (*n* = 195)
RMSE, bpm	0.46	1.13
Bias, bpm	0.08	0.39
95% upper limit of bias, bpm	0.98	2.46
95% lower limit of bias, bpm	−0.82	−1.68
Accuracy, %	99.18	91.79

**Table 3:** Continuous respiratory rate estimation performance.

Parameter	AHI < 30 (*n* = 122)	All Subjects (*n* = 195)
Total time, mins	51,410	80,567
RMSE, bpm	1.22	1.62
Bias, bpm	0.08	0.37
95% upper limit of bias, bpm	2.46	3.47
95% lower limit of bias, bpm	−2.31	−2.73
Accuracy, %	92.63	86.66

**Table 4:** Respiration rate estimation performance depending on different obstructive sleep apnea severities.

Parameter	Normal (*n* = 31)	Mild OSA (*n* = 46)	Moderate OSA (*n* = 45)	Severe OSA (*n* = 73)
Average overnight RR				
RMSE, bpm	0.64	0.36	0.39	1.74
Bias, bpm	0.01	0.06	0.15	0.93
95% upper limit of bias, bpm	0.66	0.36	0.37	1.49
95% lower limit of bias, bpm	1.29	0.75	0.87	3.84
Accuracy, %	96.77	100.00	100.00	79.45
Continuous RR measurements				
Total time, mins	13,702	19,241	18,467	13,702
RMSE, bpm	1.33	1.26	1.15	2.17
Bias, bpm	0.04	0.06	0.17	0.92
95% upper limit of bias, bpm	1.33	1.25	1.14	1.97
95% lower limit of bias, bpm	2.66	2.52	2.40	4.77
Accuracy, %	92.39	92.61	92.50	75.80

## Data Availability

Due to confidentiality reasons, it is not feasible to openly share the data.
